# Changes in home food inventories and food procurement practices during the COVID-19 pandemic

**DOI:** 10.1017/S1368980025101249

**Published:** 2025-10-06

**Authors:** Michelle C. Kegler, Cerra Antonacci, Shadé Owolabi, Ana Arana, Alexandra Morshed, Regine Haardörfer

**Affiliations:** Emory Prevention Research Center, Rollins School of Public Health, Emory University, 1518 Clifton Road NE, Atlanta, GA 30322, USA

**Keywords:** Home food environment, Food procurement, Home food inventories, COVID-19, SNAP, Food security

## Abstract

**Objective:**

To examine how home food inventories and food procurement practices changed due to the COVID-19 pandemic.

**Design:**

Cross-sectional baseline data from a randomised controlled trial of a home food environment intervention. Telephone interviews were conducted from October 2020 to December 2022.

**Setting:**

Four 2–1–1 United Way agencies in Georgia, USA.

**Participants:**

2–1–1 clients (*n* 602); 80·6 % identified as Black and 90·9 % as women. Mean age was 42·8 (sd = 11·80). The majority were food insecure (73·4 %) and received Supplemental Nutrition Assistance Program (SNAP) benefits (65·8 %).

**Results:**

A majority of participants reported smaller inventories of fresh fruits and vegetables (65·1 %) and unhealthy snacks (61·6 %) in the home relative to before COVID-19. The majority (55·8 %) also reported decreased shopping for fruits and vegetables and decreased use of fast food for family meals (56·1 %). Over half (56·2 %) started to use a food pantry, and 44·9 % started ordering groceries online due to COVID-19. A COVID-19 stressors scale was significantly associated with decreased odds of a smaller fresh fruit and vegetable inventory (OR = 0·61, CI 0·51, 0·73) and a smaller unhealthy snack inventory (OR = 0·86, CI 0·74, 0·99). COVID-19 stressors were also associated with changed food procurement practices, including increased online grocery shopping (OR = 1·19, CI 1·03, 1·37), and starting to use a food pantry (OR = 1·31, CI 1·13, 1·51).

**Conclusions:**

The pandemic had a significant impact on home food inventories and procurement practices. Understanding how major events such as pandemics affect home food environments may help to stave off negative nutritional outcomes from similar events in the future.

The COVID-19 pandemic disrupted lives in many ways, with the potential to influence home food environments and related behaviours^(1)^. Early in the pandemic, during the lockdown phase and before a vaccine was available, many restaurants were closed or limited to takeout, and shopping for groceries was associated with unknown exposures to the virus. Online grocery shopping increased^(2)^. For many, workplaces and schools were closed; remote learning and work became commonplace. As the pandemic progressed, it caused disruptions in supply chains, fuelled inflation and price increases and led to financial strain for many, leading to increased use of food pantries^(3–11)^. Government response mitigated some of these impacts with increased benefits to those in need, such as expanded child income tax credits and expanded Supplemental Nutrition Assistance Program (SNAP) and rental assistance programmes^(7,8)^. However, the pandemic’s widespread and potentially long-lasting impact on lives was indisputable.

A substantive body of research was conducted on the immediate impacts of the pandemic on eating behaviour early in the pandemic^(1)^. In a review of seventy-one studies focused on adults in the first few months of the pandemic, Johnson *et al.* reported that adult eating behaviour in the thirty countries studied changed relatively little for the majority of participants^(1)^. However, when eating behaviour did change, increases were most common, both in terms of the frequency and amount of food consumed. Reasons for changed eating behaviours included emotions, stress, boredom, time, eating with family and friends and increased exposure to food, including unhealthy but tempting foods at home. Several of these drivers stem from the home food environment which likely changed during the pandemic either socially (e.g. increased togetherness, family meals) or physically (e.g. home food inventories).

Although time at home increased for many during the pandemic^(5,6)^, along with increased exposure to the home food environment, less research has been conducted on how home food environments themselves were affected, especially beyond households with children and/or adolescents. A systematic review of fourteen studies on the impact of the pandemic on family food environments from the perspective of parents found numerous positive and negative changes^(2)^. Positive changes included less eating out, more home cooking and family meals, buying local products and increased concern for health and food quality. Negative changes included buying more snacks, shortages of certain foods and overeating. A longitudinal online study focused on adults, with data collection in April and November of 2020, found that at least some consequences of the pandemic persisted several months after the initial lockdowns, such as less eating at restaurants and higher levels of food insecurity^(12)^. Although cross-sectional, another study documented associations between changes in family food practices (e.g. shopping frequency, online ordering) and dietary quality and weight^(13)^.

The present study examines home food inventories and food procurement practices of 2–1–1 clients beyond the initial stages of the pandemic. 2–1–1 is a decentralised national information and referral service that connects people to available community resources for a broad range of topics and related social needs, such as rental and utility assistance, childcare support and food insecurity. 2–1–1 clients tend to be lower income than the general population and face a variety of social needs and daily stressors^(14,15)^. The objectives of this study were to explore how home food inventories (e.g. fresh produce, unhealthy snacks) and food procurement practices, including food shopping, fast food for family meals and food pantry usage, changed as a result of the pandemic, especially in vulnerable households. The study also examines associations between food insecurity, SNAP benefits, perceived stress, COVID-19 related impacts (i.e. increased financial challenges) and demographic characteristics and changes in the home food environment. Understanding how major stressful events such as pandemics affect nutrition-related practices may help to identify levers for reducing negative impacts in the future.

## Methods

### Study population

Data were from a hybrid-effectiveness implementation trial of the Healthy Homes/Healthy Families intervention to improve healthy eating and prevent weight gain in partnership with four United Way 2–1–1 organisations in Georgia^(16,17)^. Clients who called one of the United Way 2–1–1 organisations and expressed interest in the study were eligible to participate if they could speak English, were between the ages of 18 and 70 and reported a BMI ≥ 20 kg/m^2^; BMI was included as an eligibility criterion because the intervention tested in the parent trial emphasises weight-related behaviours. Clients assessed as in crisis by 2–1–1 staff (e.g. psychological distress), and those who were pregnant were excluded. Recruitment took place from October 2020 through December 2022. The Emory University Institutional Review Board approved the study.

### Data collection

All data were collected via telephone by trained university staff and graduate students and entered into a REDCap database. Up to twelve attempts (e.g. telephone, text, e-mail, mail) were made to reach each client referred by 2–1–1. Participants were mailed $40 via a reloadable gift card for completing baseline data collection ($20 for a home environment survey and $20 for two 24-h dietary recalls).

### Measures

COVID-specific measures were newly created by the study team given the novelty of the topic. They were designed to correspond to the home environment changes targeted by the intervention (e.g. home food inventories, food shopping and use of non-home sources for family meals).

#### COVID-19 influence on the home food environment changes

Participants were asked whether the availability of (1) fresh fruits and vegetables and (2) unhealthy snacks in the past month was less than, about the same or more than before COVID-19 (i.e. January and February of 2020). Response options of about the same or more than were collapsed into a single category for analysis.

#### COVID-19 influence on food procurement changes

Four items pertained to food procurement changes due to COVID-19. Participants were asked whether the frequency of shopping for fruits and vegetables in the past month was less than, about the same or more than before COVID-19, with responses recategorised into less than *v*. about the same or more than before COVID-19. Participants were also asked whether they started using a food pantry and started ordering groceries online due to COVID-19 (yes/no). Additionally, participants were asked whether the frequency of purchasing a meal from a fast-food restaurant in the past month decreased, stayed the same or increased since COVID-19; responses were then dichotomised either as decreased or stayed the same/increased.

#### COVID-19 stressors

Five challenges related to ways in which the COVID-19 pandemic affected participants were summed to create a COVID-19 stressors scale (range of 0–5). These challenges (yes/no) included children at home with schools or daycare closing, you or a household member laid off/furloughed from work, harder to pay rent or make house payments, harder to pay utilities and harder to afford food.

The *perceived stress scale*, which included four items with five response options ranging from never to very often, was used to measure perceived stress experienced by participants in the past month (e.g. how often have you felt difficulties were piling up so high that you could not overcome them)^(18)^.

#### Food insecurity, SNAP benefits and demographics

Experiences with food security were assessed with a valid two-item measure that asked whether, in the previous 12 months, participants worried whether food would run out before getting money to buy more and whether the food participants bought didn’t last and they didn’t have money to get more. Participants were classified as food insecure if they answered sometimes or often to at least one statement^(19)^. Demographic characteristics included age, sex, household size and children under the age of 18 living in the home, race/ethnicity, employment status, highest educational attainment, annual household income, marital status and receipt of benefits from various sources (i.e. SNAP)^(20)^.

### Analysis

Univariate and bivariate analyses (e.g. *t* test and *χ* ^2^) were conducted to characterise the study population and examine associations between demographics, food insecurity, SNAP benefits, perceived stress and COVID-19 specific stressors with changes in home food inventories and food procurement practices.

Multivariable logistic regression was conducted to analyse associations between food insecurity, SNAP benefits and stressor variables with changes in home food inventories (i.e. fruit and vegetable, unhealthy snacks) and food procurement changes due to COVID-19 (i.e. shopping for fruits and vegetables, purchasing fast-food for family meals, starting to use a food pantry, starting to order groceries online). Given that data collection took place from October 2020 through December 2022, we graphically explored the COVID-19 variables by data collection date; there were no discernible patterns over time. Despite this, we included the date of data collection in multivariable regressions as a covariate. The recruitment site was also included in the regressions as a covariate to adjust for potential clustering of the data by 2–1–1 agency and catchment area.

As it was possible that demographic variables operated as mediators or moderators of associations between our predictors and outcomes in ways too complex to disentangle in the present study, as part of the model-building process, demographic variables were entered one by one into the multivariable regressions to test for suppression (data not shown). Because we did find evidence of suppression across the demographic variables, we chose to exclude demographic variables in the final models that explored food insecurity, SNAP benefits, stressors and the home food environment.

## Results

### Description of study participants

Table 1 displays descriptive information for the 602 participants. Participants were primarily Black (80·6 %), women (90·9 %), not married (54·8 %), had children under 18 years of age in the home (59·0 %), had annual household incomes less than or equal to $25 000 (65·2 %) and participated in SNAP (65·8 %). The majority were food insecure (73·4 %). The average age was 42·8 years (sd = 11·80), nearly half of the participants were not employed (48·5 %) and 40·5 % reported a high school diploma, GED or less. The mean perceived stress score was 7·0 (sd = 3·50) of 16 on the perceived stress scale. Finally, participants averaged 3·3 (sd = 1·50, range of 0–5) on the COVID-19 stressors scale.

**Table 1.** Description of study participants (*n* 602)

	*n* or mean	% or sd
Age, mean, sd	42·8	11·80
Sex, *n*, % female	547	90·9
Household size, mean, sd	3·13	1·90
Live alone, *n*, %	124	20·6
Children < 18 in the home, *n*, % yes	355	59·0
Food security, *n*, % food insecure	442	73·4
Race/ethnicity, *n*, %		
White, not of Hispanic origin	68	11·3
Black, not of Hispanic origin	485	80·6
Hispanic	10	1·7
Asian	4	0·7
American Indian/Alaska Native	1	0·2
More than one race	24	4·0
Unknown or not reported	10	1·7
Education, *n*, %		
High school diploma, GED or less	244	40·5
Some college or technical school	222	36·9
College degree or more	136	22·6
Employed, *n*, %		
Working full-time	166	28·4
Working, part-time	103	17·6
Retired	32	5·5
Not employed/homemaker/student/on disability	283	48·5
Annual household income, *n*, %		
≤$25 000	376	65·2
$25 001–$50 000	147	25·5
$50 001–$75 000	29	5·0
> $75 000	25	4·3
Marital status, *n*, %		
Married or living with a partner	142	23·6
Widowed, separated or divorced	130	21·6
Not married	330	54·8
Receive benefits from, *n*, %		
SNAP	396	65·8
Medicaid	320	53·2
Medicare	90	15·0
Disability, unemployment, child support, alimony	201	33·4
BMI, mean, sd	33·8	8·58
Perceived stress scale^*^, mean, sd	7·0	3·50
COVID-19 stressors scale^†^, mean, sd	3·3	1·50
Influence of COVID-19 on the home food environment, *n*, % less than before COVID-19		
Fresh fruit and vegetable availability	392	65·1
Unhealthy snack availability	371	61·6
Shopping for fruits and vegetables	336	55·8
Fast food for family meals	337	56·1
Started using a food pantry, *n*, % yes	338	56·2
Started ordering groceries online, *n*, % yes	270	44·9

* Range 0–16, with higher scores indicating more stress. ^†^Range 0–5.

Table 1 also details the influence of COVID-19 on participants’ home food environments and eating behaviours. Participants reported that fresh fruits and vegetables (65·1 %) and unhealthy snacks (61·6 %) were less available in their homes compared with before COVID-19. A majority started to use a food pantry (56·2 %), and 44·9 % started ordering groceries online due to COVID-19. The majority (55·8 %) also reported decreased shopping frequency for fruits and vegetables and reductions in the use of fast food for family meals (56·1 %).

### Home food inventories

#### Fresh fruit and vegetable inventory

In bivariate analyses, fresh fruit and vegetable inventories were more likely to be smaller than prior to the pandemic for participants who were food secure, less stressed in general and by COVID-19, younger and identified as Black or White, relative to another race/ethnicity (Table 2). In the multivariable analysis, the COVID-19 stressor scale (OR = 0·61, 95 % CI 0·51, 0·73) and the perceived stress scale (OR = 0·91, 95 % CI 0·85, 0·97) were associated with decreased odds of a smaller fruit and vegetable inventory (Table 3). In contrast, those receiving SNAP benefits had increased odds of reporting a smaller fruit and vegetable inventory than prior to the pandemic, relative to those not receiving SNAP benefits (OR = 1·82, CI 1·16, 2·86).

**Table 2.** Bivariate associations between demographic characteristics and stressors with changes in home food inventories due to COVID-19

	Fresh fruit and vegetable inventory					Unhealthy snack inventory				
	Less than		The same or more than		*P*	Less than		The same or more than		*P*
	*n*/mean	%/sd	*n*/mean	%/sd		*n*/mean	%/sd	*n*/mean	%/sd	
Food security										
Food insecure	264	59·7	178	40·3	**< 0·0001**	256	57·9	186	42·1	**0·002**
Food secure	127	79·9	32	20·1		114	71·7	45	28·3	
SNAP										
No	130	63·1	76	36·9	0·46	130	63·1	76	36·9	0·59
Yes	262	66·2	134	33·8		241	60·9	155	39·1	
Stressors										
Perceived stress scale	6·4	3·43	8·0	3·41	**< 0·0001**	7·0	3·51	6·9	3·48	0·77
COVID-19 stressors scale	3·0	1·56	4·0	1·14	**< 0·0001**	3·2	1·57	3·6	1·34	**0·0006**
Demographics										
Age	42·0	11·63	44·1	12·02	**0·04**	42·5	11·63	43·2	12·08	0·46
Race										
White	43	63·2	25	36·8	**0·007**	53	77·9	15	22·1	**0·01**
Black	327	67·4	158	32·6		291	60·0	194	40·0	
Other	22	44·9	27	55·1		27	55·1	22	44·9	
Income										
≤ $25 000	235	62·5	141	37·5	0·09	222	59·0	154	41·0	**0·01**
$25 001–50 000	101	68·7	46	31·3		89	60·5	58	39·5	
$50 001–75 000	21	72·4	8	27·6		22	75·9	7	24·1	
> $75 000	21	84·0	4	16·0		22	88·0	3	12·0	
Household size	3·1	1·77	3·1	3·14	0·93	3·2	1·98	3·0	1·77	0·14
Employment										
Working full-time	117	70·5	49	29·5	0·29	105	63·3	61	36·8	0·07
Working, part-time	66	64·1	37	35·9		71	68·9	32	31·1	
Retired	20	62·5	12	37·5		23	71·9	9	28·1	
Not employed/ homemaker/student/ on disability	174	61·5	109	38·5		160	56·5	123	43·5	
Education										
High school diploma, GED or less	155	63·5	89	36·5	0·53	150	61·5	94	38·5	0·97
Some college or technical school	143	64·4	79	35·6		136	61·3	86	38·7	
College degree or more	94	69·1	42	30·9		85	62·5	51	37·5	
Marital status										
Married or living with a partner	92	64·8	50	35·2	0·71	95	66·9	47	33·1	0·23
Widowed, separated or divorced	81	62·3	49	37·7		74	56·9	56	43·1	
Not married	219	66·4	111	33·6		202	61·2	128	38·8	

Significant p-values are bolded.

**Table 3.** Adjusted multivariable associations between food insecurity, SNAP and stressors with household food inventories and food procurement practices

	Home food inventory changes				Food procurement changes							
	Less fresh fruits and vegetables		Less unhealthy snacks		Decreased shopping for Fruits and Vegetables		Started ordering groceries online		Started using a food pantry		Less use of fast food for family meals	
	OR	95 % CI	OR	95 % CI	OR	95 % CI	OR	95 % CI	OR	95 % CI	OR	95 % CI
Food security												
Food insecure (ref)												
Food secure	1·29	0·74, 2·22	1·65	0·99, 2·73	**1·77**	**1·04, 3·02**	1·20	0·74, 1·94	**0·51**	**0·31, 0·82**	1·04	0·64, 1·67
SNAP												
No (ref)												
Yes	**1·82**	**1·16, 2·86**	0·96	0·63, 1·47	**1·57**	**1·01, 2·46**	1·02	0·67, 1·53	0·91	0·59, 1·39	0·81	0·54, 1·23
Stressors												
Perceived stress scale	**0·91**	**0·85, 0·97**	1·03	0·97, 1·10	**0·92**	**0·86, 0·98**	0·97	0·92, 1·03	1·01	0·95, 1·07	0·99	0·93, 1·04
COVID-19 stressors scale	**0·61**	**0·51, 0·73**	**0·86**	**0·74, 0·99**	**0·65**	**0·55, 0·77**	**1·19**	**1·03, 1·37**	**1·31**	**1·13, 1·51**	1·12	0·97, 1·29

Significant p-values are bolded.

#### Unhealthy snack inventory

In bivariate analyses (Table 2), unhealthy snack inventories were more likely to be smaller for those who were food secure, had fewer COVID-19 stressors, identified as White and had higher annual incomes. In multivariable analyses (Table 3), a greater number of COVID-19 stressors experienced by respondents were associated with decreased odds of reporting a smaller snack inventory than prior to the pandemic (OR = 0·86, 95 % CI 0·74, 0·99).

### Food procurement practices

#### Shopping for fruits and vegetables

Bivariate analyses for food procurement changes are in Table 4. Less frequent shopping for fruits and vegetables than prior to the pandemic was more likely for those who were food secure and had lower general stress and COVID-19 stressors, as well as for younger age and those identifying as Black. In multivariable analyses, there was a positive association with food security (OR = 1·77, 95 % CI 1·04, 3·02) and receipt of SNAP benefits (OR 1·57, 95 % CI 1·01, 2·46) with decreased grocery shopping for fruits and vegetables (Table 3). There was an inverse association with the COVID-19 stressors scale (OR = 0·65, 95 % CI 0·55, 0·77), as well as with the perceived stress scale (OR = 0·92, 95 % CI 0·86, 0·98).

**Table 4.** Bivariate associations between demographics and stressors with food procurement changes due to COVID-19

	Shopping for fruits and vegetables					Started ordering groceries online					Started using a food pantry					Decreased fast food for family meals				
	Less than		The same or more than		*P*	No		Yes		*P*	No		Yes		*P*	Less than		The same or more than		*P*
	*n*/mean	%/sd	*n*/mean	%/sd		*n*/mean	%/sd	*n*/mean	%/sd		*n*/mean	%/sd	*n*/mean	%/sd		*n*/mean	%/sd	*n*/mean	%/sd	
Food security																				
Food insecure	241	54·5	201	45·5	**< 0·0001**	248	56·1	194	43·9	0·40	163	36·9	279	63·1	**< 0·0001**	254	57·5	188	42·5	0·28
Food secure	124	78·0	35	22·0		83	52·2	76	47·8		100	62·9	59	37·1		83	52·5	75	47·5	
SNAP																				
No	122	59·2	84	40·8	0·57	118	57·3	88	42·7	0·45	102	49·5	104	50·5	**0·04**	115	55·8	91	44·2	0·98
Yes	244	61·6	152	38·4		214	54·0	182	46·0		162	40·9	234	59·1		222	56·2	173	43·8	
Stressors																				
Perceived stress scale	6·5	3·57	7·8	3·20	**< 0·0001**	7·1	3·58	6·9	3·40	0·67	6·5	3·62	7·4	3·36	**0·003**	6·9	3·47	7·1	3·54	0·45
COVID stressors scale	3·0	1·58	3·9	1·20	**< 0·0001**	3·2	1·49	3·5	1·49	**0·003**	2·9	1·59	3·7	1·34	**< 0·0001**	3·5	1·41	3·2	1·59	**0·03**
Demographics																				
Age	41·8	11·61	44·3	11·94	**0·01**	44·7	12·3	40·3	10·7	**< 0·0001**	42·1	11·85	43·3	11·75	0·22	42·9	11·61	42·5	12·07	0·68
Race																				
White	37	54·4	31	45·6	**0·02**	35	51·5	33	48·5	0·79	31	45·6	37	54·4	0·15	34	50·8	33	49·3	0·41
Black	307	63·3	178	36·7		269	55·5	216	44·5		218	45·0	267	55·0		272	56·1	213	43·9	
Other	22	44·9	27	55·1		28	57·1	21	42·9		15	30·6	34	69·4		31	63·3	18	36·7	
Income																				
≤ $25 000	224	59·6	152	40·4	0·41	217	57·7	159	42·3	0·41	141	37·5	235	62·5	**< 0·0001**	217	57·7	159	42·3	0·80
$25 001–50 000	92	62·6	55	37·4		73	49·7	74	50·3		79	53·7	68	46·3		78	46·6	68	53·4	
$50 001–75 000	17	58·6	12	41·4		16	55·2	13	44·8		16	55·2	13	44·8		16	44·8	13	55·2	
> $75 000	19	76·0	6	24·0		13	52·0	12	48·0		19	76·0	6	24·0		13	48·0	12	52·0	
Household size	3·2	1·99	3·0	1·76	0·29	2·9	1·79	3·4	2·01	**0·007**	3·1	2·07	3·2	1·76	0·46	3·2	1·78	3·1	2·05	0·50
Employment																				
Working full-time	105	63·2	61	36·8	0·80	76	45·8	90	54·2	**0·02**	86	51·8	80	48·2	**0·01**	82	49·4	84	50·6	0·13
Working, part-time	62	60·2	41	39·8		55	53·4	48	46·6		49	47·6	54	52·4		54	52·4	49	47·6	
Retired	20	62·5	12	37·5		22	68·8	10	31·3		16	50·0	16	50·0		17	53·1	15	46·9	
Not employed/ homemaker/ student/on disability	166	58·7	117	41·3		167	59·0	116	41·0		104	36·8	179	63·2		170	60·3	112	39·7	
Education																				
High school diploma, GED or less	148	60·7	96	39·3	0·89	147	60·3	97	39·7	0·10	93	38·1	151	61·9	**0·03**	131	53·9	112	46·1	0·65
Some college or technical school	133	59·9	89	40·1		117	52·7	105	47·3		100	45·1	122	54·9		129	58·1	93	41·9	
College degree or more	85	62·5	51	37·5		68	50·0	68	50·0		71	52·2	65	47·8		77	56·6	59	43·4	
Marital status																				
Married or living with a partner	93	65·5	49	34·5	0·18	84	59·2	58	40·8	0·32	62	43·7	80	56·3	0·24	78	55·3	63	44·7	0·67
Widowed, separated or divorced	71	54·6	59	45·4		75	57·7	55	42·3		49	37·7	81	62·3		69	53·1	61	46·9	
Not married	202	61·2	128	38·8		173	52·4	157	47·6		153	46·4	177	53·6		190	57·6	140	42·4	

#### Starting to grocery shop online

In bivariate analyses, experiencing more COVID-19 stressors was associated with starting to grocery shop online, as were younger age and larger household size. Working full-time was also associated with starting to shop online (Table 4). In multivariable analyses (Table 3), participants had higher odds of starting to grocery shop online with higher scores on the COVID-19 stressors scale (OR 1·19, 95 % CI 1·03, 1·37).

#### Starting to use a food pantry

Starting to use a food pantry was more common among food insecure participants, those receiving SNAP benefits and among those with higher general stress and higher levels of COVID-19-related stressors. Additionally, those with lower annual household incomes and those not employed and with less education were more likely to start using a food pantry (Table 4). In multivariable analyses (Table 3), food secure participants had lower odds of starting to use a food pantry due to COVID-19 compared with participants experiencing food insecurity (OR = 0·51, 95 % CI 0·31, 0·82). Further, COVID-19 stressors were associated with increased odds of starting to use a food pantry due to COVID-19 (OR 1·31, 95 % CI 1·13, 1·51).

#### Use of fast food for family meals

In bivariate analyses, decreased use of fast food for family meals was associated with higher COVID-19 stressors (Table 4). None of the variables examined were significantly associated with the use of fast food for family meals in multivariable models (Table 3).

## Discussion

This study examined perceived changes in household food inventories and food procurement practices during the COVID-19 pandemic in a population of persons seeking assistance through 2–1–1. With respect to home food inventories, we found that over 60 % of participants perceived a decrease due to COVID-19, with food secure and those with less stress in general and specific to COVID-19 more likely to report less fruit and vegetables in the home than prior to the pandemic, as well as smaller unhealthy snack inventories. It may be that those who were food secure and less vulnerable to economic hardship, captured in our COVID-19 stressors scale, had larger home food inventories pre-COVID in contrast to food insecure persons who may have already had smaller inventories (e.g. a floor effect). Smaller home food inventories among those experiencing food insecurity are well-documented^(21–25)^. Interestingly, our findings differ from those of Adams *et al.* ^(23)^, who found that early in the pandemic, higher proportions of food insecure persons decreased their home food inventories during the pandemic, with the exception of non-perishable foods, which increased for about half of both food secure and food insecure households. Differences in our findings may be due to our longer timeframe and a possible sustained impact of the pandemic among lower-income individuals, whether food secure or not experiencing exacerbated financial burdens from the pandemic.

Perhaps not surprisingly, shopping for fruits and vegetables followed the same general pattern, with food secure persons and those with less stress more likely to report decreased shopping frequency due to COVID-19 than those who were food insecure and more stressed. Again, there may have been more room for changes in shopping behaviour for food secure households as some research has found more frequent grocery shopping trips per month among food secure households^(26)^. However, our findings contradict results by Baxter *et al*.^(27)^, who found that shopping more frequently compared with the same frequency before the COVID-19 pandemic was associated with greater odds of food insecurity, though our samples and methods differed in several meaningful ways (e.g. rurality, data collection methods, food insecurity time frame).

Another major finding was that over half of the participants started to use a food pantry due to COVID-19. This was more common among those who were food insecure and among those with more COVID-19 stressors. This is consistent with national reports of increased food pantry use given exacerbated economic hardship for many during the pandemic^(9,10)^. Additionally, over half of the participants decreased their use of fast food for family meals, which is consistent with several other studies documenting decreased fast food, restaurant food and/or takeout during the pandemic^(13,28,29)^. Consistent with other studies, starting to order groceries online was also influenced by COVID-19, but among less than half of the participants^(13,29)^. Those with greater COVID-19 stressors were more likely to report starting to order groceries online.

SNAP benefits were expanded during the pandemic, and this study explored whether benefits may have helped to mitigate the negative influences of the pandemic on household food inventories and practices. Interestingly, we found that SNAP recipients were more likely to report smaller fruit and vegetable inventories as a result of the pandemic than were those not receiving SNAP, and SNAP benefits were also associated with increased use of food pantries. This suggests that while SNAP likely was helpful, it was not sufficient to stock households with an adequate food supply, particularly one that included fruits and vegetables.

This study has several limitations, including its cross-sectional nature, which precludes examinations of causality. Exploratory analyses with the demographic variables in regression models suggested that the demographic variables were involved in mediation with the predictors and outcomes. Qualitative and longitudinal research is needed to better understand the causal relationships between individual characteristics, stressors and the home food environment. Additionally, the data are self-reported and collected over a 26-month period, resulting in different reference frames with respect to the start of the pandemic. Finally, generalisability may be limited given that study participants were recruited from 2–1–1 agencies and represent a unique population facing numerous challenges, even without the additional stress of a global pandemic.

### Conclusion

The pandemic had a significant impact on home food environments, with smaller fresh fruit and vegetable inventories, decreased fruit and vegetable shopping, decreased use of fast food for family meals and a significant increase in food pantry usage. Future research could examine these types of changes to home food environments and food procurement behaviours longitudinally to assess their impact on long-term changes in home food patterns. These findings may also inform efforts that warrant evaluating (e.g. mobile markets or home delivery of local produce, healthier food available at food pantries, financial incentives for healthier food) to mitigate potential negative nutrition-related impacts in future stressful events.
